# Impact of a Revised Curriculum Focusing on Clinical Neurology and Musculoskeletal Care on a Required Fourth-Year Medical Student Physical Medicine and Rehabilitation Clerkship

**DOI:** 10.1155/2016/6197961

**Published:** 2016-11-29

**Authors:** John W. Norbury, Clinton E. Faulk, Kelly M. Harrell, Luan E. Lawson, Daniel P. Moore

**Affiliations:** ^1^Department of Physical Medicine and Rehabilitation, Brody School of Medicine at East Carolina University, Greenville, NC, USA; ^2^Department of Anatomy and Cell Biology, Brody School of Medicine at East Carolina University, Greenville, NC, USA; ^3^Department of Academic Affairs, Brody School of Medicine at East Carolina University, Greenville, NC, USA

## Abstract

*Background*. A Required Fourth-Year Medical Student Physical Medicine and Rehabilitation (PM&R) Clerkship was found to increase students' knowledge of PM&R; however the students' overall rotation evaluations were consistently lower than the other 8 required clerkships at the medical school.* Objective*. To describe the impact of a revised curriculum based upon Entrustable Professional Activities and focusing on basic pain management, musculoskeletal care, and neurology.* Setting*. Academic Medical Center.* Participants*. 73 fourth-year medical students.* Methods*. The curriculum changes included a shift in the required readings from rehabilitation specific topics toward more general content in the areas of clinical neurology and musculoskeletal care. Hands-on workshops on neurological and musculoskeletal physical examination techniques, small group case-based learning, an anatomy clinical correlation lecture, and a lecture on pain management were integrated into the curriculum.* Main Outcome Measurements*. Student evaluations of the clerkship.* Results*. Statistically significant improvements were found in the students' evaluations of usefulness of lecturers, development of patient interviewing skills, and diagnostic and patient management skills (*p* ≤ 0.05).* Conclusions*. This study suggests that students have a greater satisfaction with a required PM&R clerkship when lecturers utilize a variety of pedagogic methods to teach basic pain, neurology and musculoskeletal care skills in the rehabilitation setting rather than rehabilitation specific content.

## 1. Introduction

Joint disorders and spine pain are the first and third most common primary diagnoses for physician office visits, respectively [1], yet these conditions are often underrepresented in medical education in both the clinical and preclinical years [2, 3]. While there are very few required Physical Medicine and Rehabilitation (PM&R) clerkships in the United States [4], such experiences can both be meaningful for students and improve their knowledge of pain, musculoskeletal, and neurological disorders [5, 6].

In 2014, the American Association of Medical Colleges (AAMC) developed a list of core Entrustable Professional Activities (EPAs) for Entering Residency. The 13 EPAs comprise a list of “activities that all entering residents should be expected to perform on day one of residency without supervision” [7]. While conditions commonly encountered in PM&R are not mentioned by name in the EPAs, the first three EPAs are to perform a history and physical examination, prioritize a differential diagnosis, and recommend and interpret common diagnostic tests. Research has suggested that medical students feel less comfortable performing these activities in patients with musculoskeletal and neurological disorders than other medical diagnoses. For example, a study at Harvard Medical School in 2007 found that medical students did not feel adequately prepared to practice musculoskeletal medicine and lacked “clinical confidence” and “cognitive mastery” in the field [2]. Another study has demonstrated a lack of confidence among medical students with regard to patients who have neurological complaints to the point where a term “Neurophobia” has been coined in the literature [8]. Finally, most primary care providers do not feel comfortable managing patients with chronic, nonmalignant pain [9] and recent Centers for Disease Control and Prevention (CDC) guidelines have emphasized the importance of nonopioid treatment for chronic pain [10]. Proposals for integration of PM&R into the four-year medical student curriculum [11, 12] and for a required clerkship in PM&R [4] have been put forth, in part, as ways to address these knowledge deficits among medical students. It has been demonstrated that a required PM&R clerkship can improve students' musculoskeletal physical examination skills [6].

We sought to improve the quality of the two-week required clerkship in PM&R during the fourth-year of a primary care medical school curriculum. The rotation was established in 2009 in response to a curricular deficiency in care of patients with neurologic and musculoskeletal conditions. A previous study demonstrated that this rotation increased the students' knowledge of PM&R and enhanced their clinical skills [5]. Despite these findings, the clerkship was consistently ranked lowest among the eight required clerkships at the medical school by the students in terms of overall student satisfaction with the rotation.

The goal of the curricular changes was to improve the educational experience through a focus on the subset of PM&R clinical knowledge and skills a primary care oriented intern physician would need in clinical neurology and musculoskeletal care when viewed through the lens of the EPAs. By shifting the focus of the rotation from rehabilitation toward the neurological and musculoskeletal conditions most commonly encountered in primary care, we hoped to better align the clerkship with the mission of the medical school which includes training primary care providers.

In the 2014-2015 academic year, a lecture on the musculoskeletal and neurological physical examination was provided on the first day of the clerkship. The students then participated in bedside teaching rounds where an attending physician accompanied the medical students to interview and examine patients with the following diagnoses: stroke, brain injury, limb deficiency, back pain, and spinal cord injury. They rotated through general and specialty inpatient rehabilitation units as well as a variety of outpatient settings during the two-week rotation (Table 1). The students attended two departmental grand rounds and two resident didactic lectures given by PM&R faculty on rehabilitation topics. A more detailed description of the structure of the clerkship prior to the curricular changes has been described previously [5].

## 2. Methods

Based on anonymous student feedback and clerkship evaluations, quality improvement methodology was used to identify opportunities for educational improvement that were instituted in July 2015. The assigned readings were changed from modified excerpts of the American Academy of Physical Medicine and Rehabilitation (AAPM&R) study guides to selections from* Current Medical Diagnosis and Treatment (CMDT) 2015 *[13] covering musculoskeletal care and selected neurology topics. This textbook was chosen as it is a highly rated reference used by primary care providers and also freely available to the students as an e-book through the university's library. The CMDT readings focused more on the diagnosis, workup, and treatment of common neurological and musculoskeletal conditions, rather than rehabilitation aspects of these disease processes which was the focus of the previous readings. Two hands-on 60-minute physical examination workshops were added on the neurologic and musculoskeletal physical examination. The neurological examination included a review of the assessment of strength, sensation, and muscle stretch reflexes based upon the American Spinal Cord Injury (ASIA) physical examination with an emphasis on utilizing the neurologic exam to localize spinal cord and peripheral nerve lesions. The musculoskeletal examination workshop discussed and demonstrated the approach to the shoulder, elbow, hand, knee, and ankle examination. A two-hour case-based workshop was also added, which consisted of three cases (hand pain, shoulder pain, and back pain) incorporating principles of the neurological and musculoskeletal clinical evaluation, differential diagnosis, workup, and treatment of these common clinical conditions. All of these lecturers were led by resident physicians under direct real time faculty supervision. In addition, a 60-minute interactive, case-based lecture, conducted by a faculty member in the Department of Anatomy and Cell Biology, was integrated into the rotation. This lecture provided students with a focused review of the anatomy pertinent to common primary care musculoskeletal and neuromuscular disorders. Finally, a 60-minute “chalk-talk” by a PM&R faculty member was added addressing pain management, bowel management, and bladder management issues that are most commonly encountered by residents on day one of intern year. The practice examination and clerkship final examination (both 30-question multiple-choice examinations) were revised to encompass the updated content.

During the period of the study the clinical rotation schedule did not change and the students continued to participate in 2 hours of departmental grand rounds and 2 hours of resident lectures. The students still had inpatient and outpatient experiences each of which was approximately 50% of the clinical time. With the addition of the didactic activities above, the overall amount of didactic time increased from 7.5 hours (9.4% of the clerkship) to 12.5 hours (15.6% of the clerkship).

The curriculum changes described above were approved by the Brody School of Medicine Clinical Curriculum Committee and Executive Curriculum Committee prior to implementation in July 2015. First semester 2014 rotation evaluations were compared to first semester 2015 rotation evaluations using a Mann–Whitney *U* test. The evaluations were submitted by the students at the end of the rotation via the *E* ^*∗*^ value (http://www.evaluehealthcare.com/) electronic evaluation system. Qualitative, anonymous medical student comments regarding the strengths and weaknesses of the rotation were reviewed after the implementation of the changes. This study was determined to be exempt from IRB approval by East Carolina University Institutional Review Board.

## 3. Results

**Figure 1:**
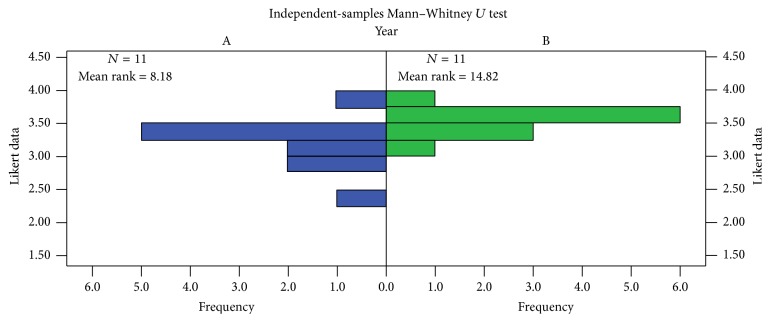
Mann–Whitney *U* test results for 2014 versus 2015. Group A = 2014; Group B = 2015.

The rotation evaluations were completed by 36 students in 2014 and 37 students in 2015. Because these evaluations were required for completion of the course, the response rate was 100%. Since there were two unpaired samples with an ordinal outcome measure, a Mann–Whitney *U* test was used to compare the overall course evaluations between 2014 and 2015 (Figure 1). A statistically significant (*p* < 0.05) difference was found between the two groups (*p* = 0.016). We further compared the individual questions on the rotation evaluations from the fall semesters before and after the curricular changes (Table 2). Four statistically significant questions (*p* < 0.05) were identified related to clearly specified learning objectives, usefulness of lecturers, development of patient interviewing skills, and diagnostic and patient management skills. Selected comments regarding strengths and opportunities for improvement with regard to the educational content in the rotation following the changes are shown as follows:

*Selected Excerpts from Medical Student Comments in the 2015 Rotation Evaluation*

*Strengths of the Rotation*

“I learned how to complete a more thorough neuro and MSK exam. I learned a lot more about the different levels of dermatomes, myotomes, etc. and how to put all of that information together in a differential. THIS WAS INVALUABLE!!”

“Great in depth review of musculoskeletal and neuro that rotations 3rd year skimmed over.”

“I enjoyed increasing my knowledge on how to complete a full and expansive neurologic exam and musculoskeletal complaints.”

“Love that [the lecturer] really focused on the primary care and neurology issues that we will see in the future. The PM&R information was interesting but not always applicable to what we plan to do in the future. I appreciate that [the lecturer] took the parts that were applicable and taught us valuable tips for intern year.”

*Specific Ways the Rotation Could Be Improved*

“Would enjoy ‘a what is PM&R' lecture that talks about the various things they do and common reasons for referral for people entering other fields.”

“More presence before 4th year.”

“I actually would recommend that this would be a 3rd year course as I think it would help with [USMLE] Step 2 [Clinical Skills].”

“Some of the resident didactics were over our heads at the M4 level. The time might be better spent reading or in a lecture for our level of training.”

## 4. Discussion

The curricular changes described above had a statistically significant positive effect on students' rating of a required two-week PM&R clerkship for fourth-year medical students. Of note, the domains where the improvements were statistically significant were clinical aspects of the rotation that were specifically targeted by the revised curriculum (learning objectives, lecturers, and patient evaluation and management skills). This suggests that the improved performance was related to the curricular changes rather than a global improvement in the rotation such as better resident teachers or more experienced faculty in the 2015 academic year compared to 2014. During the time period of our study the clerkship director, clerkship coordinator, PM&R faculty, department chairperson, department administrator, and clinical experiences remained consistent, suggesting these were not confounding variables.

The students themselves commented in their evaluations how helpful it was to learn clinical neurology and musculoskeletal care in the rehabilitation setting. One student specifically noted how the focus on neurology and musculoskeletal care that they will encounter in practice was appreciated even though they found the rehabilitation topics interesting. While many physiatrists may enjoy teaching topics such as prosthetics or musculoskeletal ultrasound, these topics may not be common enough in general practice to warrant devoting large amounts of precious curricular “real estate” to them in a two-week required PM&R rotation for all medical students. Indeed one student commented that the PM&R resident lectures they did attend were “over our heads at the M4 level.” This highlights the importance of teaching to the learner's level of training in PM&R education. Other rehabilitation topics such as stroke, low back pain, and concussion are rather common in general practice and students appreciated the “PM&R perspective” on the diagnosis and management of these common conditions. This “anchored learning” environment where students are exposed to content that they are likely to encounter in practice can facilitate efficient and effective learning [14]. With ever increasing amounts of knowledge, a required PM&R rotation that is not grounded in core skills needed by all physicians runs the risk of contributing to “curriculomegaly.” In this condition, students are exposed to ever-increasing amounts of information crowded into a limited amount of time [15]. The authors have found the EPAs to be a very useful tool for PM&R medical school curriculum designers to focus on teaching core skills to students in a meaningful clinical context. Finally, PM&R is the ideal specialty to address the diagnosis and management of chronic pain conditions such as fibromyalgia and the psychosocial aspects of pain in general. We covered these topics in the lecture on pain, bowel, and bladder management.

**Figure 2:**
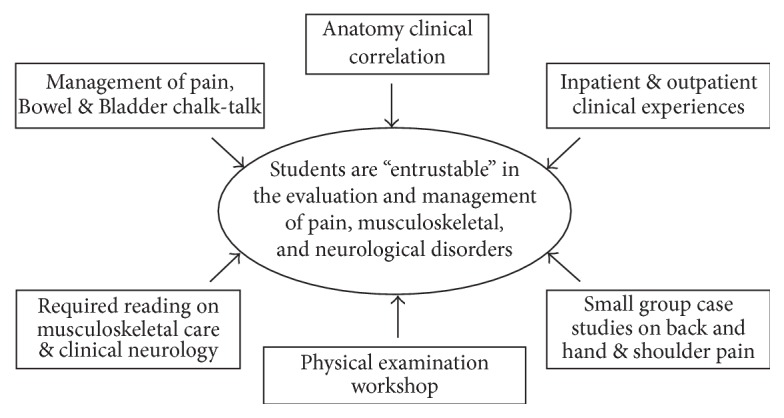
Conceptual framework for the revised two-week required PM&R clerkship for fourth-year medical students.

Since this rotation is the last required component of the medical school curriculum at our institution devoted to neurological and musculoskeletal disorders, the PM&R clerkship represents the capstone where the pluripotent medical student codifies the clinical skills necessary to manage these conditions at the level of an intern physician. A conceptual framework for how the clerkship seeks to accomplish this goal is provided in Figure 2.

Basic science is an essential aspect of clinical care that has historically been taught during the first two years of medical school; however there are benefits to integration of the basic sciences throughout all four years of medical school [16, 17]. Incorporation of basic science into PM&R clerkship via anatomy clinical correlation didactics provides a model which may be helpful for medical schools that wish to revisit basic science in the clinical years. When anatomy is presented at this stage in medical education, the clinical relevance is more readily appreciated (e.g., the difference between carpal tunnel syndrome and a C6 radiculopathy and the components of the rotator cuff). A required PM&R clerkship provides an excellent opportunity to review anatomy and other basic science topics such neurophysiology, pharmacology, and pathology in a clinical context that strengthens understanding and long-term retention.

Another important benefit of the curricular changes is that the resident physicians at our institution now have several dedicated opportunities to teach medical students which enhances their educational experience and improves compliance with item IV.A.5.c.(8) of the ACGME Program Requirements for Physical Medicine and Rehabilitation [18]. Direct observation of resident teachers also allows the Residency's Clinical Competency Committee to better assess a resident's progression through the Practice-Based Learning and Improvement Milestone 1 [19]. It has previously been demonstrated that utilizing PM&R residents as teachers in musculoskeletal care is well received by the learners [20].

A required PM&R rotation certainly has the potential to target other skills needed by medical graduates beyond what we have done in the present study. Resident physicians must be able to give or receive handover to transition care responsibilities (EPA 8) [7]. Fourth-year students may have a better ability to execute a handoff than students earlier in their training and an inpatient PM&R service may be a good venue to practice and assess this skill. Students are expected to “collaborate as members of an interprofessional team” (EPA 9) [7]. The very nature of PM&R includes interdisciplinary care and there are opportunities to foster collaboration with allied health professionals such as having students lead patient care conferences.

## 5. Study Limitations

Limitations of our study include the fact that the outcomes data were based on medical student opinions rather than more objective measures of knowledge gained such as written examinations, United States Medical Licensing Examination (USMLE) examination scores, and Objective Structured Clinical Examinations (OSCEs). Unfortunately, the change in the required readings necessitated a new final rotation examination which prevented comparison of the students' medical knowledge before the intervention and that after the intervention. Also, the overall amount of lecture time was increased, so the improvement in rotation feedback may have been a reflection of the number of hours of teaching rather than improved quality or content of teaching. However, none of the subjective comments specifically mentioned the lecturers added in 2015 as an area for improvement and 9 students (24%) specifically referenced one or more of the new didactic sessions as a strength of the rotation.

## 6. Conclusions

This study suggests that students have a greater satisfaction with a required PM&R clerkship when lecturers focus on primary care oriented basic clinical neurology and musculoskeletal care skills in the rehabilitation setting using a variety of pedagogic methods. EPAs may be a helpful tool for PM&R medical school curriculum designers who wish to integrate PM&R into medical student education.

## Figures and Tables

**Table 1:** Clinical rotations in the PM&R Clerkship (2014–2016).

Inpatient experiences	Outpatient experiences	
*Required*	Electrodiagnosis (EMG)	Spinal cord injury
General rehabilitation	Pain management	Mild traumatic brain injury
Consults	Musculoskeletal ultrasound	Wheelchair
*Selected by student (1 of 3)*	Spasticity management	Acupuncture
Traumatic brain injury	Wound care	Pediatric rehabilitation
Spinal cord injury	Occupation medicine	Amputee
Pediatrics	Spine injections	General rehabilitation

**Table 2:** Medical student-reported first semester rotation performance, 2014 versus 2015.

Item number	Description	2014 mean	2015 mean	*p*
1	Overall rotation rating	2.83	3.25	0.062
2	Learning objectives were clearly specified	3.28	3.65	**0.014**
3	Didactic sessions were useful	3.14	3.54	**0.022**
4	The method of determining grades was fair	3.28	3.62	0.153
5	The experience facilitated development of my patient interviewing skills	2.94	3.32	**0.031**
6	The experience enhanced my diagnostic and patient management skills	3.00	3.49	**0.011**
7	The rotation director provided me with mid- rotation feedback	2.48	3.13	0.283
8	Supervising physicians were available for discussions regarding patient care	3.33	3.70	0.526
9	I was encouraged to use the latest medical evidence	3.33	3.53	0.368
10	The overall quality of residents' teaching on this clerkship was high	3.33	3.65	0.078
11	The rotation complied with duty hours and on-call requirements	3.89	3.92	0.665

Rating scale: Question 1: 0, poor; 1, fair; 2, average; 3, very good; 4, excellent. Questions 2–11: 1, almost never; 2, sometimes; 3, usually; 4, almost always.
